# Evaluating the feasibility and effectiveness of a capacity-building model to nurture junior independent clinical research investigators in Uganda

**DOI:** 10.1371/journal.pone.0335299

**Published:** 2026-07-24

**Authors:** Aidah Nanvuma, Joseph Musaazi, Adelline Twimukye, Vivian Nakate, Agnes Kiragga, Miriam Laker-Oketta, Christine Sekaggya-Wiltshire, Yukari C. Manabe, Barbara Castelnuovo

**Affiliations:** 1 Infectious Diseases Institute, College of Health Sciences, Makerere University, Kampala, Uganda; 2 Johns Hopkins University, Maryland, Baltimore, United States of America; 3 African Population and Health Research Center, Nairobi, Kenya; 4 Innovations for Poverty Action, Kampala, Uganda; 5 University of Antwerp, Belgium; King Abdulaziz University Faculty of Medicine, SAUDI ARABIA

## Abstract

**Background:**

Research capacity-building initiatives remain crucial to achieving Sustainable Development Goal 3 on health and well-being, especially in LMICs. We aimed to evaluate the effectiveness of the Infectious Disease Institute’s (IDI) Capacity-Building Model to nurture junior independent clinical research investigators in Uganda.

**Methods:**

From 13^th^ July 2021–06^th^ February 2023, we conducted a cross-sectional study using a mixed-methods approach to assess the extent to which the Capacity-Building Model was effective, feasible, and acceptable. For quantitative research, we conducted an online survey with 80 scholars (Master’s, PhD, and Post-doctoral fellows), comprising 20 alumni and 60 current scholars, to explore their experiences and perceptions as former and current scholars of the Capacity Building Unit (CBU) at IDI. For qualitative research, we purposively selected 20 scholars to participate in the in-depth interviews.

**Results:**

Participants reported that the capacity-building Model had a beneficial impact on their career progression, with 90% expressing a willingness to recommend it to others. The overall scientific benefit reported was 48.7%; this was significantly higher among continuing scholars than among alumni (56.7% vs 25.0%, respectively; p-value = 0.046). Additionally, 85% achieved their career goals, and 65% said it expanded their employment opportunities. Qualitative findings highlighted its significant positive impact on research training and professional development. Participants praised the Model’s emphasis on mentorship, with both scientific and non-scientific support proving crucial in guiding junior researchers through technical challenges, manuscript writing, and career planning. Soft skills training, dissemination platforms, and networking opportunities further contributed to scholars’ academic growth. Scholars benefited from robust institutional support, including access to research infrastructure, grantsmanship assistance, and administrative systems. However, challenges such as limited research funding, slow procurement processes, and delays in supervision hindered progress. The COVID-19 pandemic also disrupted mentorship and training. Participants recommended improvements in mentorship coordination, procurement efficiency, broader model visibility, and expansion to other universities and disciplines to enhance its effectiveness and sustainability.

**Conclusion:**

Overall, the Capacity-Building Model was highly acceptable among scholars; however, minor administrative challenges need to be addressed to further enhance learning. We recommend tailored, relevant scholarly programs to meet the evolving needs of emerging scientists and foster scholarly growth and research innovation in academic institutions, enabling them to tackle local public health challenges. Future research is required to assess the cost of capacity building and its sustainability.

## Introduction

### Background

Research capacity-building remains crucial for improving health outcomes in resource-constrained settings [1]. This entails intentionally developing sustainable individual and institutional [2,3] research capacity-building skills, structures, and resources to achieve the Sustainable Development Goals (SDGs), particularly SDG 3 on Good Health and Well-Being [4]. However, formal research training in higher education institutions remains limited [5]. Training is usually complemented by mentorship. Mentorship involves providing guidance, knowledge, and support from an experienced individual, referred to as a mentor, to a less experienced individual, known as a mentee, to help them navigate a career pathway [6,7]. The primary objective of mentorship relationships is to enable the mentor and mentee to acquire skills in the proper conduct of research, dissemination of research results, and knowledge management and utilization. Prior studies report two common forms of mentorship: peer-to-peer and senior-to-junior mentorship [8]. With peer-to-peer mentorship, students or colleagues with similar experiences, ages, and levels of power engage in reciprocity by offering psychosocial support and facilitating learning exchanges with one another [9,10]. The senior-to-junior format may involve an experienced faculty member mentoring a less experienced member to transfer skill sets and knowledge.

Mentorship remains fundamental in nurturing the next generation of researchers, as valuable knowledge and expertise are passed down, thereby strengthening research capacity. Moreover, developing professional networks facilitates collaboration and knowledge sharing among researchers locally and globally [9]. These networks provide valuable opportunities for researchers to exchange ideas, collaborate on projects, and access resources and expertise beyond their immediate environments [10].

Despite notable progress in health research capacity development in LMICs, there remains substantial room for improvement [5,10]. Few programs focus on investing in or implementing systemic or institutional-level approaches to capacity development [5,11]. We therefore reflected on the operational aspects of a Sustainable Research Capacity Building Model used to nurture credible researchers at the Infectious Diseases Institute (IDI), Uganda [12], to solicit research scholars’ experience and appraisal of our systems and infrastructure towards nurturing independent research investigators.

## Methodology

### Study design

From 13^th^ July 2021–06^th^ February 2023, we conducted a cross-sectional study using a mixed-methods approach to evaluate the effectiveness, feasibility, and acceptability of the Capacity-Building Model used at IDI. For the quantitative component, a structured online survey was administered to Master’s, PhD, and post-doctoral scholars, including both alumni and current scholars, to explore their experiences and perceptions of the Capacity Building Unit (CBU) programs. Additionally, we conducted 20 in-depth interviews with participants across all training levels to gain a deeper understanding of their experiences. For this study, alumni were defined as scholars who had completed the training program and successfully published at least one peer-reviewed manuscript arising from their program-supported research. Continuing scholars were defined as those who were still actively enrolled in the program and had not yet achieved this publication milestone, regardless of their program duration. This distinction was based on programmatic status and progression rather than academic seniority, with publication serving as a pragmatic indicator of program completion and transition. A mentor is a researcher with a postgraduate qualification, at least five years of independent research experience, and a proven record of publications, mentorship, grant acquisition, and research leadership. A post-doctoral scholar is a researcher who has completed a PhD and is engaged in further advanced research training to deepen expertise, expand their research portfolio, and transition toward independent research leadership. A PhD (Doctor of Philosophy) scholar is an advanced-level trainee engaged in doctoral training focused on generating original research that contributes new knowledge to a specific field. A Master’s scholar is an early-stage postgraduate trainee undertaking structured academic and research training to develop foundational research skills. Data collection took longer than expected due to the COVID-19 interruption in 2021.

### Study setting

The IDI was established in 2002 under the College of Health Sciences at Makerere University. It is a Ugandan not-for-profit organization dedicated to strengthening Africa’s health systems, focusing on infectious diseases through research and capacity development [11,13]. IDI comprises six departments: Research, Prevention Care and Treatment, Health System Strengthening, Training, Global Health Security, and Laboratory Services. The IDI Research Department CBU has supported over 100 research scholars at the Post-doctoral, research fellowship, Ph.D., and Master’s levels by providing mentorship paired with economic and infrastructural support under the leadership of the Head of the Research Department [14].

### Description of the capacity building model at IDI

The IDI established a formal and structured Capacity Building Unit in 2016 to strengthen research capacity among young investigators and early-career scientists. The unit was designed as a coordinated institutional intervention to address gaps in research training, mentorship, and career development and to foster a sustainable, locally led research ecosystem.

**Fig 1:**
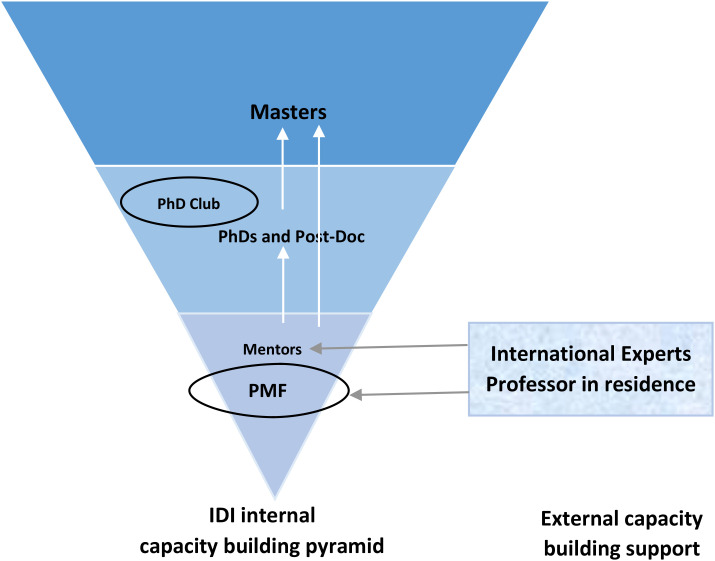
Shows the sustainable capacity-building model at IDI. Legend: Arrows indicate mentorship from senior to Junior supporting evaluations and individual students. Circles indicate peer mentorship. A mentor is a researcher with a postgraduate qualification, at least five years of independent research experience, and a proven record of publications, mentorship, grant acquisition, and research leadership.

The Model is a multi-layered, mentorship-driven framework grounded in two core principles: vertical mentorship from senior to junior scientists and horizontal peer-to-peer mentorship (Fig 1). The Model integrates scientific training, mentorship, and enabling institutional support across career stages, including Master’s scholars, PhD scholars, post-doctoral fellows, and senior scientists. All scholars formally enrolled in IDI-supported research training programs had access to the Model and actively participated in its core components as part of their fellowship or training requirements.

The scientific components of the Model focus on strengthening research knowledge, skills, and scholarly independence [15]. These include structured individual mentorship and supervision, in which each scholar is paired with one or more senior mentors who guide research activities and career development. Learning opportunities are provided through short courses, research forums, soft skills training, and regular scholar evaluations, covering areas such as epidemiology, data analysis, Good Clinical Practice, research ethics, grant writing, manuscript development, and scientific communication. In addition, the Model incorporates structured peer mentoring through forums that bring together senior scientists, emerging scientists, PhD scholars, research fellows, and social scientists, where participants present proposals and findings, review manuscripts, discuss literature, and share methodological and professional expertise.

**Fig 2:**
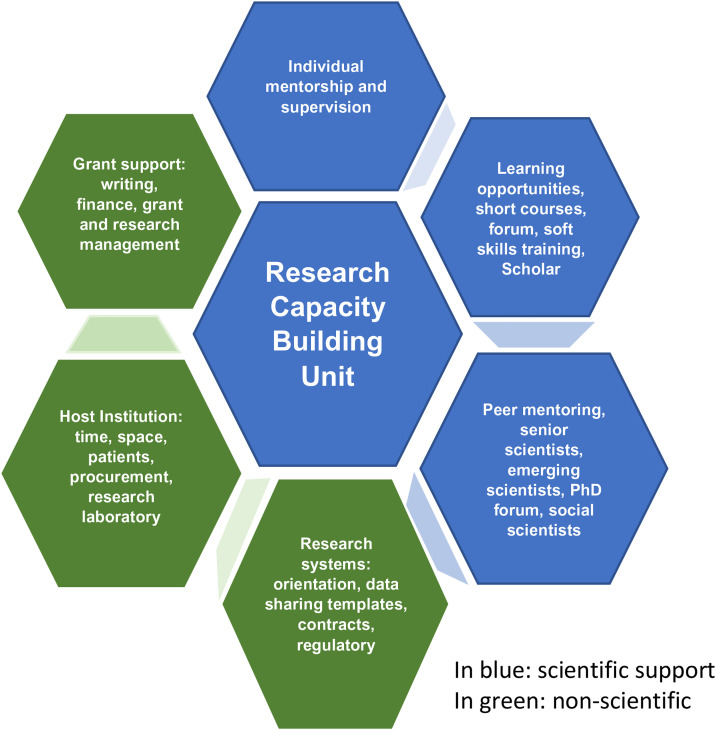
Scientific and non-scientific support provided by the Capacity Building Unit to research trainees at IDI.

Complementing the scientific components, the Model includes non-scientific support mechanisms that create an enabling research environment and reduce operational barriers. These include institutional research systems such as structured orientation, data-sharing mechanisms, standardized templates, contract management processes, and regulatory and ethics support. Scholars also benefit from host institution support, including protected research time, access to physical space, clinical datasets, procurement systems, and research laboratories. In addition, comprehensive grants and research management support is provided, encompassing grant writing assistance, financial management, and grants administration (Fig 2).

At the time the Model was implemented in 2016, mentorship at IDI relied heavily on international experts. Over time, mentorship has deliberately transitioned to locally led senior scientists, many of whom are IDI alumni who were themselves trained under international mentorship. This evolution reflects increasing institutional maturity and sustainability. International experts continue to play a complementary role by advising senior scientists and providing advanced technical input in specialized areas where local expertise or infrastructure remains limited, such as laboratory sciences.

### Participant selection and sampling

Study participants for the quantitative survey were approached by email to provide consent to participate in the interviews. We received questionnaire responses from 80 scholars in the capacity-building unit, comprising alumni and continuing scholars. We included all scholars affiliated with IDI CBU since its inception who were available and provided consent to participate in the study. For the qualitative component, we conducted 20 in-depth interviews drawn from the 80 participants who completed the quantitative questionnaire. Interviewees were purposively selected across three categories: Masters (8), PhD (7), and post-doctoral (5). Interviews within each group were conducted until thematic saturation was achieved, as determined through analysis of detailed field notes and debrief summaries. Participant lists were generated from CBU records and included contact information such as email addresses and telephone numbers.

### Data collection

We conducted an online survey using Kobo Toolbox. An email with a link to the online Informed Consent Form and the survey questionnaire was sent to all participants to solicit their participation. To increase the response rate, follow-up emails and telephone call reminders were sent to participants who had not completed the survey within 1–2 weeks of the online questionnaire response invitation. Data were collected using a pre-coded questionnaire administered in English. The questionnaire was carefully designed to comprehensively capture participants’ experiences with the Capacity Building Unit and ensure clarity and consistency in responses. Information collected included a) socio-demographic characteristics. b) academic, professional engagement and achievements before, during, and after their participation in the unit. c) scientific and non-scientific support based on their perceived contribution to achieving expected scholarly outputs, and provide brief explanations of their rankings. d) Challenges encountered: Scholars described challenges faced at different stages of training, highlighting obstacles that may have affected their learning or progression. e) Programmatic gaps and recommendations: Data were collected on perceived gaps in the unit’s programs and on suggestions for improvement, providing insights into how the unit could enhance its support for scholars. f) post-training attitudes and perceptions among alumni participants, enabling a comprehensive assessment of the program’s impact.

A Likert scale from 1 to 5 will be used for the survey questions to rank the contribution of unit support activities to scholarly outputs. Where; 1 = Strongly Disagree, 2 = Disagree, 3 = Neutral (Undecided), 4 = Agree, and 5 = Strongly Agree. Participants could only submit their responses after completion. For qualitative data, we conducted in-depth interviews with Master’s, Ph.D., and post-doctoral scholars. We collected data using a topic guide to assess participant experiences, challenges, gaps, and recommendations for unit programs and activities.

For qualitative analyses, we assessed alumni and continuing scholars’ experiences, attitudes, and beliefs on post-training and current engagements through in-depth interviews to explore in greater depth and capture nuanced insights into the Capacity Building Unit until thematic saturation was reached. Semi-structured interview guides were used, with prompts designed to explore participants’ backgrounds, motivations for joining the unit, and their experiences with specific Unit activities and programs. These included both scientific support and non-scientific support. Participants were asked to reflect on highlights and positive experiences during their time in the unit, as well as on challenges encountered. They were also asked to suggest solutions to these challenges and provide recommendations for improving participation in current or future Unit activities at individual, community, institutional, or national levels. Interviews were conducted in English, audio-recorded, and lasted 30–45 minutes. All participants provided written informed consent before participation. The data collection period spanned from July 2021 to February 2023, during which interviews were transcribed verbatim and systematically analyzed to identify key themes related to alumni development, program effectiveness, and areas for improvement.

### Definition of primary outcome

The primary outcome of the study was scientific benefits, which included 1.) Individual mentorship and supervision, 2.) Learning opportunities: short courses, forums, soft skills training, and scholar evaluations, and 3.) Peer mentoring, Senior scientists, emerging scientists, PhD forum, and Social Scientists. Non- Scientific support includes: 1.) Research systems: orientation, data sharing, templates, contracts, and regulatory support. 2.) Host Institution: Time, space, patients, procurement, research laboratory. 3.) Grants Support: writing, finance, grants, and research management.

Overall score for scientific benefit was obtained from 15 question items from 13 to 27 in the continuing Scholars’ questionnaire and 13–27 in the Alumni’s questionnaire (supplementary files 1 and 2) using a 5-level Likert scale (−2 = Strongly disagree, −1 = Disagree, 0 = Neutral, 1 = Agree, 2 = Strongly Agree). Total score classified as High (15–30), Moderate (10–14), and Low (−30–9). Quantitative data in the results stem from the questionnaire, while the qualitative data come from the in-depth interviews

### Data management and analysis

Quantitative data were extracted from NVIVO Toolbox (https://www.kobotoolbox.org/) and analyzed using Stata v.15.1 (StataCorp, College Station, TX, USA). At analysis, participants were categorized into alumni and continuing scholars, and the results were summarized using frequencies and percentages.

An inductive thematic approach was used for data analysis, focusing on qualitative data derived from a topic guide. Three themes were identified from the questionnaire analysis, including the effectiveness and approaches of the capacity-building Model, challenges or gaps in the Model, and recommendations.

Researchers reviewed transcribed in-depth interviews to familiarize themselves with the data. A joint coding framework was developed from five transcripts representing 25% of the total to enhance consistency and transparency in coding. Two coders (AT and JB) performed open coding. Initial codes were refined through consensus and consultation with the Principal Investigator, and reliability was ensured by cross-checking among coders. The transcripts were organized and imported into NVivo version 12 (QSR International Pty Ltd., 2018) for open coding, resulting in code categorization and theme identification. A final merged codebook synthesized key findings, and illustrative quotations were selected to represent each theme in the results.

The study was approved by The AIDS Support Organisation Research Ethics Committee (accreditation number: UG-REC-009). The study also received approval from the UNCST (study reference number: HS1100ES). Informed consent was obtained from each participant before their responses to the questionnaire and interviews. Administrative clearance was obtained from IDI to carry out this study.

## Results

### Social demographic characteristics of the study participants

We invited 100 participants, of whom 80 responded to the online survey, yielding a response rate of 80%. Of the 80 participants enrolled, 20 were alumni (Masters: 17, PhD: 2, Post-doc: 1) and 60 continuing research scholars (Masters: 39, PhD: 13, Post-doc: 8) (Table 1). The median age of alumni was 35 years (IQR: 30.5–42.0), and 34 years for continuing scholars (IQR: 29.5–38.0). 66% were males (Alumni: 65.0%; Continuing: 66.7%).

**Table 1:** Social demographics participant characteristics.

Variable	Alumni scholars (20, 25%) n (%) or median (IQR)	Continuing scholars (60, 75%) n (%) or median (IQR)	Total (80, 100%) n (%) or median (IQR)
**Age in years (** *Median [IQR]* **)**	35 (30.5 - 42.0)	34 (29.5 - 38.0)	34 (30-38)
**Education level completed or pursuing.**			
Masters	17 (85.0)	39 (65.0)	56 (70.0)
PHD	2 (10.0)	13 (21.7)	15 (18.8)
Post Doc	1 (5.0)	8 (13.3)	9 (11.3)
**Sex**			
Male	13 (65.0)	40 (66.7)	53 (66.3)
Female	7 (35.0)	20 (33.3)	27 (33.8)
**Years of experience After First Degree**			
<=5 years	5 (25.0)	22 (36.7)	27 (33.8)
>5 years	15 (75.0)	38 (63.3)	53 (66.3)

IQR stands for Interquartile range; n, number of participants; % percentage; experience after first degree refers to the total professional experience accumulated by participants after completing their first academic degree, regardless of whether it was in research or clinical practice. This includes research-related roles, clinical work, teaching, program management, or other professional positions in health and related fields.

### Feasibility and effectiveness of the capacity-building model

#### Capacity building model effectiveness.

**Fig 3:**
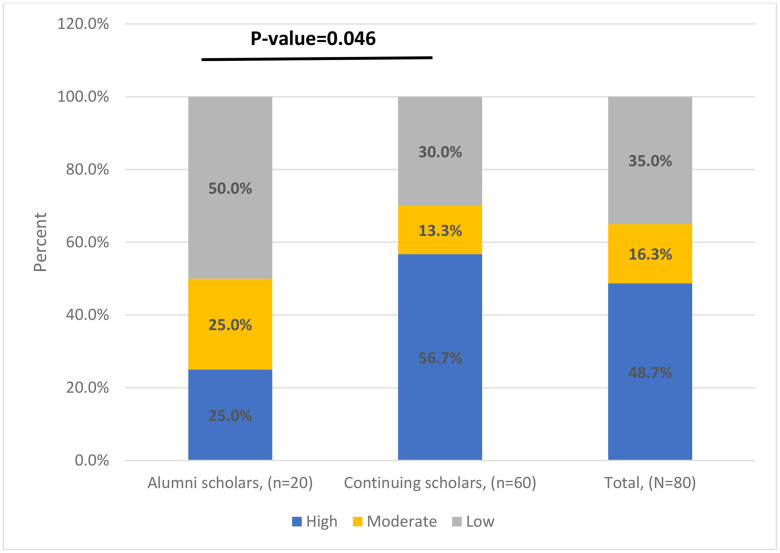
Perceived scientific benefits as scored by alumni and continuing scholars.

Participants reported that the capacity-building Model had a beneficial impact on their career progression, with 90% expressing a willingness to recommend it to others. Overall, 48.7% of respondents reported high perceived scientific benefits; this was significantly higher among continuing scholars compared to alumni (56.7% vs 25.0%, respectively, p-value = 0.046; Fig 3)

#### Approaches to the capacity building model.

Participants described several capacity-building approaches that significantly supported their academic growth, with mentorship and support supervision emerging as central pillars. Respondents emphasized that individual scientific and non-scientific mentorship offered critical technical guidance, regular progress evaluation, and constructive feedback on research manuscripts. This mentorship was seen as instrumental in helping them acquire the skills and tools needed for academic and professional advancement. Many scholars have highlighted the value of a supportive supervision model that responds to their needs in real time, facilitating faster progress in their research. They expressed appreciation for mentors who possessed deep knowledge and leadership experience, noting these qualities as key to meaningful guidance. In addition to technical mentorship, participants recognized the importance of soft skills training, including writing, communication, and critical thinking, which they felt contributed to their overall development as well-rounded researchers. They also reported that research dissemination platforms, such as weekly forums and journal clubs, exposed them to diverse perspectives and opened the door to collaboration. Support from the research office, particularly in terms of ethical review processes and grantsmanship, was also noted as crucial in helping them navigate complex academic environments. Networking events and targeted workshops were credited with fostering collaboration and idea exchange. At the same time, the strong research infrastructure at the IDI was repeatedly mentioned as a foundational resource that enabled effective implementation of research activities Table 2.

**Table 2:** Scientific and non-scientific support provided by the capacity building unit to IDI alumni and continuing scholars.

	Continuing Scholars Frequency (%), n = 60					Alumni Frequency (%), n = 20				
Questionnaire prompts¶	Strongly agree	Agree	Neutral	Disagree	Strongly disagree	Strongly agree	Agree	Neutral	Disagree	Strongly disagree
**Scientific Support**										
13. I attribute the success of my research and career development to individual mentorship and supervision received from the Unit	25 (41.7)	24 (40.0)	7 (11.7)	3 (5.0)	1 (1.7)	5 (25.0)	10 (50.0)	5 (25.0)	–	–
14. I attribute the success of my research and career advancement to the learning opportunities received from the unit (short courses, soft skills)	24 (40.0)	18 (30.0)	12 (20.0)	5 (8.3)	1 (1.7)	5 (25.0)	9 (45.0)	5 (25.0)	1 (5.0)	–
15. I attribute the success of my research and career advancement to peer mentorship groups provided by the Unit (E.g. Emerging scientist, social scientist, and PhD club and senior scientist forum)	14 (23.3)	22 (36.7)	17 (28.3)	5 (8.3)	2 (3.3)	3 (15.0)	5 (25.0)	7 (35.0)	5 (25.0)	–
19. Learning opportunities such as soft skills training, short courses (online/offsite/onsite), John Hopkins Summer Institute and Professor in Residence programs had the greatest impact on my research and career success.	18 (30.0)	25 (41.7)	14 (23.3)	3 (5.0)	–	4 (20.0)	7 (35.0)	6 (30.0)	2 (10.0)	1 (5.0)
20. Research dissemination platforms such as Research Forum and journal club had the greatest impact on my research and career success.	23 (38.3)	21 (35.0)	12 (20.0)	4 (6.7)	–	6 (30.0)	8 (40.0)	5 (25.0)	1 (5.0)	–
21. The evaluation sessions such as Bi- annual evaluations and quarterly reports submissions were useful to my research and career advancement.	22 (36.7)	24 (40.0)	13 (21.7)	1 (1.7)	–	4 (20.0)	7 (35.0)	6 (30.0)	3 (15.0)	–
**Non-scientific Support**										
16. I attribute the success of my research and career advancement to the Grants support (Writing, finance, grants, and research management) received	18 (30.0)	18 (30.0)	13 (21.7)	7 (11.7)	4 (6.7)	2 (10.0)	10 (50.0)	7 (35.0)	–	1 (5.0)
17. IDI as a host institution provided sufficient support (Space, patients cohorts data, procurement, and research lab) for my research studies	28 (46.7)	26 (43.3)	6 (10.0)	–	–	4 (20.0)	11 (55.0)	4 (20.0)	1 (5.0)	–
18. I received the support about Research systems at IDI (Orientation, data sharing templates, Contracts and regulatory) for my studies	25 (41.7)	21 (35.0)	10 (16.7)	4 (6.7)	–	6 (30.0)	6 (30.0)	6 (30.0)	2 (10.0)	–
**Capacity Building Model**										
22. The Capacity Development Unit model used to nurture an independent investigator at IDI was relevant for my research training needs	29 (48.3)	25 (41.7)	6 (10.0)	–	–	5 (25.0)	10 (50.0)	5 (25.0)	–	–
23. The Capacity Building Unit model used to nurture independent investigators was effective for my research and training needs	25 (41.7)	24 (40.0)	11 (18.3)			3 (15.0)	10 (50.0)	5 (25.0)	2 (10.0)	–
24. The Capacity Building Unit model used to nurture independent investigators at IDI was efficient for my research training and needs.	25 (41.7)	22 (36.7)	12 (20.0)	1 (1.7)	–	3 (15.0)				
25. The Capacity Building Unit model used to nurture independent investigators at IDI is sustainable.	17 (28.3)	32 (53.3)	9 (15.0)	2 (3.3)	–		10 (50.0)	5 (25.0)	2 (10.0)	–
26. The Capacity Building Unit model used to nurture independent investigators at IDI is consistent.	21 (35.0)	28 (46.7)	10 (16.7)	1 (1.7)	–	4 (20.0)	11 (55.0)	4 (20.0)	1 (5.0)	–
27. The Capacity Building Unit model used to nurture independent investigators at IDI is coherent and well-coordinated.	23 (38.3)	27 (45.0)	9 (15.0)	1 (1.7)	–	5 (25.0)	11 (55.0)	3 (15.0)	1 (5.0)	–

¶ question prompts are picked from the Alumni and continuing scholars’ questionnaires (supplementary file 1 and 2) tables summarize questions and responses directly from the study questionnaires.

Overall, perceptions of the Capacity Building Unit were strongly positive among continuing scholars (n = 60) and alumni (n = 20).

For scientific support, 81.7% of continuing scholars and 75.0% of alumni credited mentorship for their research and career success, while 70.0% in both groups valued learning opportunities. Research dissemination platforms were endorsed by 73.3% of continuing scholars and 70.0% of alumni.

For non-scientific support, 90.0% of continuing scholars and 75.0% of alumni reported sufficient institutional support. Grants support received 60.0% agreement in both groups, with some disagreement noted among continuing scholars (18.4%).

The overall capacity building model was highly rated, with 78–90% of continuing scholars and 65–80% of alumni agreeing that it was relevant, effective, consistent, and well-coordinated.

#### Mentorship as an accelerator of scholarly development.

Mentorship emerged as a central mechanism through which scholars acquired technical competence, research confidence, and professional identity as expressed. *“Although I did not have a formal international mentor, being part of broader research networks exposed me to global expertise and technical guidance”* (Post-doc, Male). Beyond routine supervision, mentorship functioned as an enabling structure that reduced uncertainty, accelerated progress, and integrated scholars into wider academic networks. The responsiveness and accessibility of mentors, particularly their respect for time and commitment to feedback were critical in sustaining momentum. Importantly, mentorship extended beyond formal supervisor, mentee dyads to include informal and international networks, suggesting that exposure to distributed expertise compensated for the absence of direct international supervision. Scholars’ emphasis on mentors’ expertise, leadership, and research credibility reflects the symbolic role mentors played in legitimizing scholars’ academic trajectories.

#### Learning opportunities through soft skills development.

Soft skills training was perceived as foundational to scholars’ holistic development, bridging the gap between technical knowledge and effective scholarly practice. *“The soft skills training significantly improved my writing and how I present data, which strengthened the quality of my work”* (Masters, Male). Skills such as scientific writing, data presentation, communication, and critical thinking enhanced scholars’ capacity to translate research outputs into publishable and fundable work. These competencies were viewed not merely as supplementary but as empowering tools that enabled scholars to navigate academic spaces more confidently and independently. The inclusion of time management and mentorship skills further suggests a deliberate effort to cultivate self-regulated and future-ready researchers.

#### Research findings dissemination as a capacity-building platform.

Regular research forums and journal clubs functioned as critical learning spaces where scholars engaged in scholarly dialogue, peer critique, and knowledge exchange. These platforms demystified the research process by exposing scholars to different stages of study development and dissemination as expressed by participant *“The weekly forums allowed us to learn from others’ studies while understanding the full research process”* (Masters, Male). The iterative feedback received during presentations supported conceptual refinement and strengthened analytical thinking. Participation in these forums also fostered academic confidence and positioned scholars as active contributors within the research community rather than passive scholars as illustrated *“Presenting at journal clubs built my confidence and strengthened my research capacity”* (Masters, Male).

#### Institutional support structures as enablers of research quality.

Support structures provided by the research office such as internal scientific reviews, ethical approvals, and administrative clearances played a formative role in shaping research rigoras illustrated, “*Each review stage added new insights and strengthened my proposal, showing that strong systems were in place”* (PhD, Male). These systems institutionalized quality assurance and encouraged iterative learning through structured critique. Scholars interpreted repeated reviews not as barriers but as developmental checkpoints that progressively refined their work. This reflects the role of institutional governance in transforming novice researchers into methodologically rigorous scholars.

#### Grantsmanship support and research sustainability.

Grantsmanship support emerged as both a practical and strategic intervention that enhanced scholars’ research independence. Access to grant-writing guidance, financial planning support, and protected research time enabled early-career researchers to conceptualize and pursue competitive funding. One post doc stated, *“The stipend and protected time allowed me to focus on grant writing and develop as an early-career researcher”* (Post-doc, Female). This support was especially critical for post-doctoral scholars transitioning toward research autonomy. The availability of stipends and time relief reduced financial pressure and allowed scholars to focus on long-term scholarly productivity.

#### Networking opportunities and professional integration.

Networking opportunities facilitated through workshops, referrals, and conferences expanded scholars’ professional capital as stated *“Being connected to experienced researchers expanded my network and provided practical guidance.”* (PhD, Female). Engagement with experienced researchers enabled informal mentorship, peer learning, and exposure to diverse career pathways. These interactions fostered a sense of belonging within the research ecosystem and enhanced scholars’ readiness to collaborate across disciplines and institutions, including at the international level.

#### Infrastructure and research ecosystem as productivity drivers.

The availability of a robust research infrastructure positioned the Institution as a self-sustaining research environment, as one participant stated, *“Access to statisticians helped validate my analysis and strengthened my confidence.”* (Masters, Male). Access to statistical expertise, digital resources, reliable internet, and institutional libraries enabled scholars to independently manage complex research tasks. These resources reduced operational barriers, enhanced analytical rigor, and improved the quality of research outputs. The institutional ecosystem was therefore perceived as integral to cultivating a culture of excellence and productivity, as a post-doc stated. *“The research ecosystem provided all the resources needed to practice high-quality research”* (Post-doc, Male).

### Challenges in the capacity-building model

#### Inadequate research funding.

Insufficient research funding emerged as the most significant constraint affecting scholars’ progress, as stated by a master’s scholar, *“My study required patient recruitment and laboratory work, but additional funding was unavailable”* (Masters, Female). Limited financial resources restricted data collection, experimentation, and access to essential materials. This financial insecurity heightened stress, reduced publication potential, and threatened career sustainability, particularly for scholars without supplementary income. The mismatch between program expectations and available funding undermined the intended impact of capacity-building efforts.

#### Procurement delays as structural barriers.

Procurement inefficiencies disrupted research timelines and reduced productivity, according to a master’s student. *“The procurement process was lengthy and delayed access to essential research equipment”* (Masters, Male). Lengthy approval processes delayed access to essential equipment, undermining time-sensitive research activities. These delays had cumulative effects, including missed deadlines and increased psychological strain, highlighting how administrative bottlenecks can disproportionately affect early-career researchers.

#### Supervisory delays and fragmented guidance.

Delayed and inconsistent supervisory feedback constrained scholarly progress and decision-making, as a PhD scholar reported, *“Receiving feedback from multiple supervisors was slow and often inconsistent, delaying progress”* (PhD, Female). Scholars with multiple supervisors experienced conflicting guidance, leading to prolonged revisions and uncertainty. This fragmentation weakened research coherence and discouraged innovation, as scholars hesitated to advance without consensus.

#### COVID-19–related disruptions.

The COVID-19 pandemic disrupted mentorship, training schedules, and mobility opportunities. Travel restrictions and laboratory closures delayed research activities and limited hands-on learning. The shift to remote engagement weakened mentorship interactions and intensified anxiety regarding career progression and funding competitiveness.

#### Scholar-related challenges.

Table 3 shows that challenges were more frequently reported during the pre and in-training periods. Among alumni (n = 20), 30.0% reported pre-training challenges, 35.0% reported challenges during training, and only 15.0% reported post-training challenges. The majority (60.0%) disagreed that they experienced challenges after graduation. Among continuing scholars (n = 60), 56.7% reported challenges before joining the unit. However, during training, fewer scholars (26.6%) reported challenges, while 48.4% disagreed that they experienced challenges. Overall, reported challenges decreased during and after engagement with the Capacity Building Unit.

**Table 3:** Challenges reported by scholars.

Variable	Responses				
Alumni respondents (N = 20)	Strongly agree	Agree	Neutral	Disagree	Strongly disagree
	n (%)	n (%)	n (%)	n (%)	n (%)
28. I encountered scholar-related challenges at IDI capacity building unit (pre-training)	–	6 (30.0)	3 (15.0)	8 (40.0)	3 (15.0)
29. I encountered scholar related challenges at IDI capacity building unit (In-training)	1 (5.0)	6 (30.0)	4 (20.0)	8 (40.0)	1 (5.0)
30. I encountered scholar related challenges (post-training) after graduating for IDI Capacity Building unit	1 (5.0)	2 (10.0)	5 (25.0)	10 (50.0)	2 (10.0)
**Continuing Scholars (N = 60)**					
28. I encountered scholar related challenges before joining the Capacity Building Unit at IDI	9 (15.0)	25 (41.7)	16 (26.7)	7 (11.7)	3 (5.0)
29. I encountered scholar related challenges while under the Capacity Building Unit at IDI	5 (8.3)	11 (18.3)	15 (25.0)	22 (36.7)	7 (11.7)

Stands for N, the total number of participants, n, the number of participants, and % for the percentage.

### Program values, gaps, and recommendations

**Fig 4:**
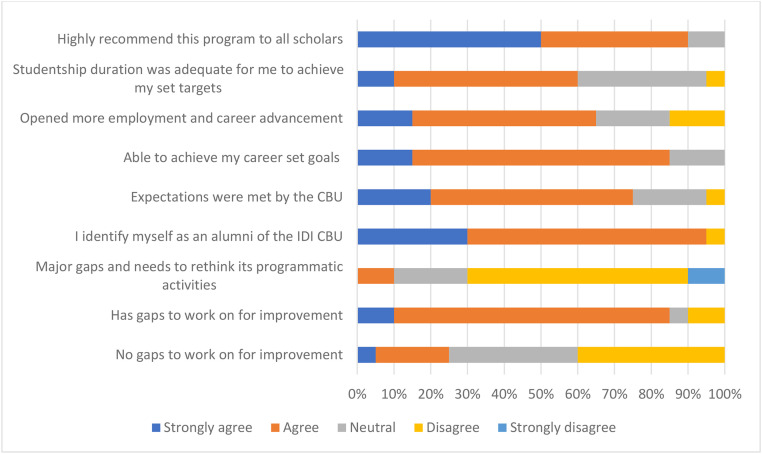
Unit values, gaps, and recommendations rating by alumni. **Legend**: IDI denotes Infectious Diseases Institute, CBU Capacity Building Unit. Percent (%) represent proportion of Alumni responses. Values: Studentship duration was adequate for me to achieve my set targets, opened more employment and career advancement, Able to achieve my career set goals, Expectations were met by the CBU, I identify myself as an alumni of the IDI CBU Gaps: Major gaps and needs to rethink its programmatic activities, has gaps to work on for improvement, no gaps to work on for improvement. Recommendations: Highly recommend this program to all scholars.

Overall, alumni reported high levels of satisfaction with the program. 90% indicated they would highly recommend the program to other scholars, and 95% identified themselves as program alumni. Most respondents reported achieving their set goals (85%) and felt that their expectations were met by the CBU (75%). The program was perceived to have opened employment and career advancement opportunities for 65% of participants, while 60% considered the studentship duration adequate. Notably, 85% identified gaps requiring improvement, highlighting opportunities for targeted program strengthening (Fig 4).

### Participant recommendations

#### Publicization and visibility of the capacity-building model.

Participants emphasized that clearer, broader communication about the capacity-building Model would enhance accessibility, inclusivity, and interdisciplinary engagement, as noted: *“Clear online information would help scholars from different disciplines understand the program”* (Masters, Male). Increased visibility was seen as essential for attracting diverse scholars, improving orientation, and strengthening stakeholder investment.

#### Scaling and multi-disciplinary expansion.

Scaling the Model beyond a single institution was viewed as a strategy to strengthen national capacity. One post-doc emphasized, *“Expanding the program to other universities would strengthen research capacity nationally”* (Post-doc, Male). Expanding to other universities and disciplines would promote equity, decentralize expertise, and foster context-specific research solutions. A multi-disciplinary approach was also seen as critical for addressing complex health and policy challenges, as stated: *“Engaging policymakers throughout the research process would improve impact”* (Post-doc, Male).

#### Strengthening mentorship coordination and procurement systems.

Improved mentorship coordination and streamlined procurement systems were identified as essential structural reforms. Scholars suggested*, “Stronger coordination among supervisors would reduce delays and confusion”* (Post-doc, Male) and *“Dedicated procurement processes for scholars would minimize time-bound disruptions”* (PhD, Male). Clear leadership, harmonized supervision, and scholar-specific procurement pathways were seen as mechanisms to reduce inefficiencies, frustration, and time loss.

## Discussion

The results of our study provide insight into the multifaceted experiences and perceptions of alumni and continuing scholars affiliated with the Capacity Building Unit (CBU) at IDI. Amidst the challenges, such as procurement delays, supervisory issues, and limited research funds, a resounding affirmation of the CBU’s invaluable support in nurturing scholars’ research and career development journeys emerges. From personalized mentorship to immersive learning experiences, peer collaboration platforms, and grant support, the CBU has emerged as a beacon of support for scholars navigating the complex landscape of academic and research endeavors. These findings align with those of Mremi et al. (2023), who highlighted that structured mentorship and institutional support are crucial for enhancing the skills, confidence, and career development of young researchers in resource-limited settings. This suggests that comprehensive mentorship models, coupled with professional and institutional support, are critical for developing independent investigators [9]. Our sustainable capacity-building Model at IDI is based on peer-to-peer and senior-to-junior mentorship approaches. Our scholars highlighted individual mentorship and supervision, training, and learning opportunities through short courses, statistical and publication support, research dissemination and evaluation meetings, grants application support, access to institutional resources, networking with renowned research experts, and additional funding for their research projects as benefits of belonging to the program.

We see a similar model by Balandya et al., which utilizes the same vertical and horizontal mentoring strategies, creating a mentored hierarchical pyramid crucial for the succession of research expertise. Similarly, scholars reported achieving substantial milestones, including publications, participation in research short-course training, enrolment in Ph.D programs, involvement in ongoing research projects, and grant applications [16].

Additionally, the “Experience after first degree” refers to the total professional experience accumulated by participants after completing their first academic degree, including roles in research, clinical practice, teaching, program management, and other health-related positions.

Notably, a high proportion of continuing scholars had ≥ 5 years of professional experience but no peer-reviewed publications, indicating that many participants entered the Capacity Building Unit with substantial practical and professional experience, even in the absence of formal research outputs. Our findings align with previous research, which emphasizes the pivotal roles of mentorship, access to resources, and institutional backing in fostering the growth and success of scholars [17,18]. Our study extends the discourse by uncovering nuanced differences in perceptions between alumni and continuing scholars. While alumni reminisce fondly about their transformative experiences at CBU, continuing scholars offer constructive feedback, highlighting areas for improvement and refinement within the program. This disparity underscores the dynamic nature of capacity-building initiatives and the imperative of ongoing evaluation and adaptation to meet evolving needs [17].

Despite the benefits mentioned, several challenges scholars encounter include inadequate research funding and stipends, procurement delays, supervisory delays, and COVID-19 interruptions, similar to those reported in other studies. The COVID-19 disruptions led to delays in data collection, slower and more expensive procurement of research supplies, and fewer physical meetings with the research team due to social distancing measures implemented during the pandemic. Our findings indicate a general reduction in scholar-related challenges faced by continuing scholars and alumni during and after their tenure at the CBU compared to before joining the unit. Nevertheless, a notable number of respondents reported challenges, particularly during the pre- and in-training phases of training under the CBU. This is consistent with Manabe et al. (2011), who observed that early-career researchers in Africa often face initial obstacles such as navigating complex research environments, accessing mentorship, and building independent research skills, but these challenges tend to decrease as trainees gain experience and structured support from capacity-building programs [6].

This indicates that while the CBU may have mitigated some challenges through streamlining the mentorship structure, providing relevant training opportunities, and supporting economic infrastructure, there are still areas, such as additional research funding, procurement, and delays in supervisory feedback, where improvement may be needed to support scholars throughout their academic journey. The study’s findings highlight the pivotal role of mentorship, access to resources, and institutional support in fostering the growth and success of scholars. A supportive environment that fosters conducive learning, collaboration, and an innovation ecosystem, such as the IDI CBU, plays a critical role in shaping the trajectory of research scholars’ careers and catalyzing scientific advancement.

Among the institutional recommendations suggested were surveys to identify barriers to career growth, allowing mentees to choose their own mentors, identifying and supporting promising students from medical schools, improving procurement processes, offering short-term fellowships at the Institute, providing adequate research funding and stipends, and strengthening peer group leadership and enrollment. This finding is consistent with evidence from Tanzania, where a unifying research mentorship model comprising senior researchers and early- and mid-career scholars in higher education institutions emphasized the importance of an enabling environment to foster collaboration, resource mobilization, training, career development, and mentorship as essential components for sustainable research output in institutions of higher learning in LMICs [9].

### Strength

This study employs a mixed-methods approach, combining quantitative and qualitative data to triangulate its findings. It further paves the way for future research avenues to deepen our understanding of capacity-building initiatives and their enduring impact on scholars’ trajectories. Longitudinal investigations tracking participants over extended periods could offer insights into the sustained efficacy of the CBU and illuminate the factors contributing to long-term success.

### Limitation

The reliance on self-reported data introduces the possibility of response bias, necessitating cautious interpretation of the findings.

## Conclusion

Our findings underscore the importance of sustained investment in research capacity-building programs and highlight the enduring impact of mentorship, collaboration, and institutional support in fostering scientific excellence and innovation. The study’s findings will serve as a benchmark for continuous quality improvement of the capacity-building process at our Institution.

## Supporting information

S1 File Survey questionnaire for IDI Capacity Building Unit continuing scholars. (PDF)

S2 File Survey questionnaire for IDI Capacity Building Unit Alumni. (PDF)
