# Correction: Evaluating the feasibility and effectiveness of a capacity-building model to nurture junior independent clinical research investigators in Uganda

**DOI:** 10.1371/journal.pone.0357299

**Published:** 2026-08-28

**Authors:** The PLOS One Staff

There are errors in the legend of Fig 1. Please see the complete, correct Fig 1 legend here.

The publisher apologizes for the error.

**Fig 1:**
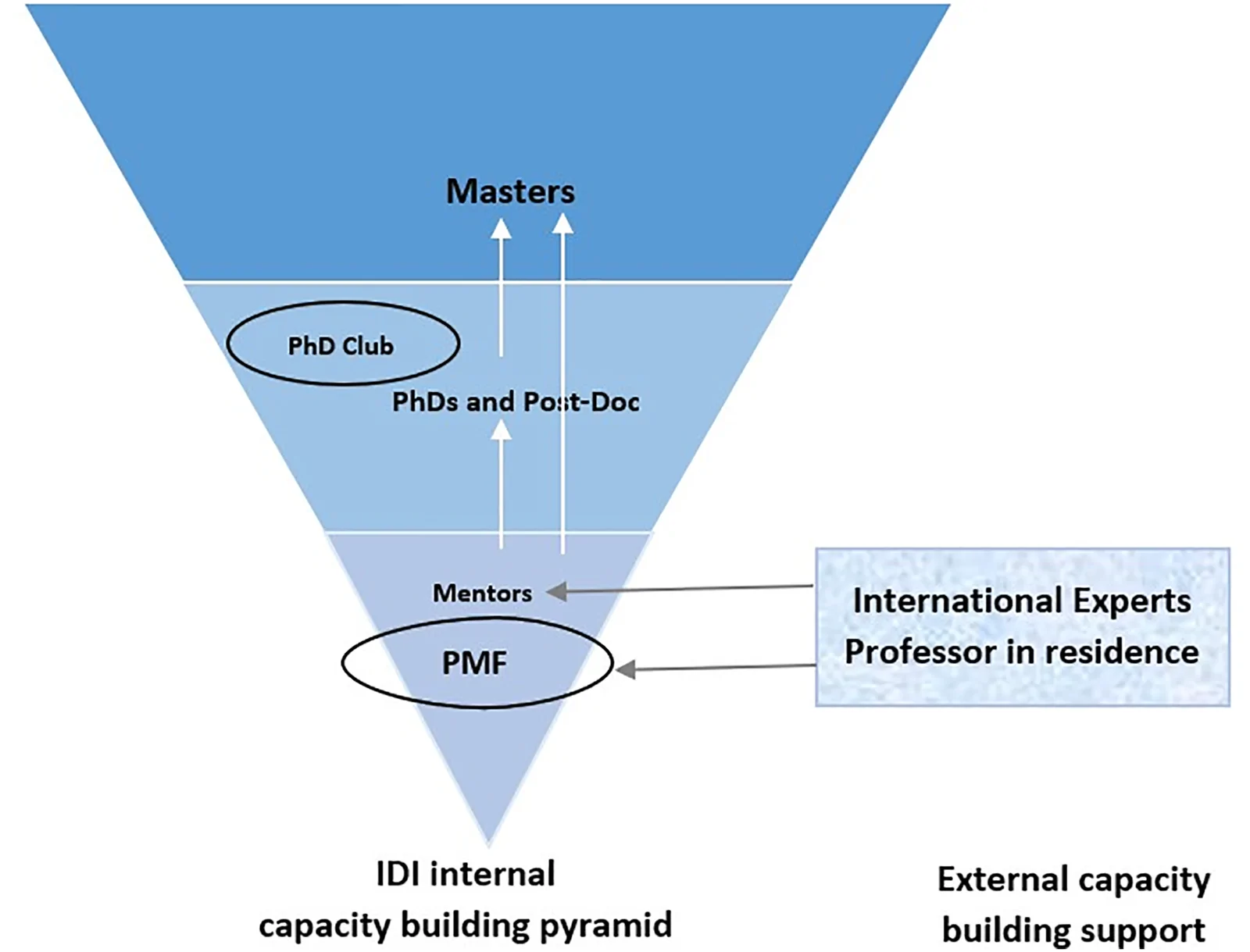
Shows the sustainable capacity-building model at IDI. Arrows indicate mentorship from senior to Junior supporting evaluations and individual students. Circles indicate peer mentorship. PMF denotes Peers Mentoring Forum. A mentor is a researcher with a postgraduate qualification, at least five years of independent research experience, and a proven record of publications, mentorship, grant acquisition, and research leadership.
