# Supplementary material for: Evaluating the feasibility and effectiveness of a capacity-building model to nurture junior independent clinical research investigators in Uganda
Source: PLoS One. 2026 Jul 24;21(7):e0335299. doi: 10.1371/journal.pone.0335299 (PMC13399504; doi:10.1371/journal.pone.0335299)
Supplement: S1 File — (PDF) [file pone.0335299.s001.pdf]

# SURVEY QUESTIONNAIRE FOR IDI CAPACITY BUILDING UNIT CONTINUING SCHOLARS

## EVALUATING THE CAPACITY BUILDING MODEL USED TO NURTURE INDEPENDENT RESEARCH INVESTIGATORS AT THE INFECTIOUS DISEASES INSTITUTE, UGANDA

## SOCIO-DEMOGRAPHICS.

1. **What is your age bracket?**
    - 18-39
    - 40-59
    - 60+

2. **Please select your gender?**
    - Male
    - Female

3. **What is your marital status?**
    - Single (Never Married before)
    - Married
    - Divorced
    - Widowed
    - Others please specify: ...........................................................

4. **Please select the best option that fully describes your profession**
    - Physician
    - Medical Officer
    - Pharmacist
    - Nursing Officer
    - Laboratory Personnel
    - Social scientist
    - General services
    - Other

5. **What is your Level of experience?**
    - 1 year
    - 2-4 years
    - 5-7 years
    - 8+ years

6. **Please select your current qualifications**
    - Masters
    - PhD
    - Post Doc

7. **What is the best description of your work place, please select all that apply and indicate if it is a public or private sector?**
    - Hospital
    - Research based institution

THE AIDS SUPPORT ORGANISATION
28 JAN 2022
Approved, Valid Until:...........
RESEARCH ETHICS COMMITTEE

- University/Teaching Institution
- Other (Specify): ………………………………………………………………

8. **Job security; Please select all what apply**
   - Short-term contract
   - Permanently employed
   - Volunteer
   - Other(Specify): ……………………………………………………………

## PROFESSIONAL ENGAGEMENTS AND ACHIEVEMENTS

9. **What activities are you engaged in? Please select all that apply**
   - Teaching activities
   - Clinical activities
   - Research activities
   - Research activities
   - Leadership and administrative roles
   - Other activities
10. **How many students are you mentoring?**
    - None
    - 1
    - 2-5
    - 6-10
    - 11+
11. **How many publications do you have?**
    - None
    - 1
    - 2-5
    - 6-10
    - 11+
12. **How many grants have you written and won?**
    - None
    - 1
    - 2-5
    - 6-10
    - 11+

THE AIDS SUPPORT ORGANISATION
28 JAN 2022
Approved, Valid Until:..........
RESEARCH ETHICS COMMITTEE

## SCIENTIFIC SUPPORT

13. **I attribute the Individual mentorship and supervision received to my research and career progress.**
    - Strongly agree
    - Agree
    - Neutral
    - Disagree
    - Strongly Disagree

14. **I attribute the learning opportunities (Short courses, soft skills) received to my research and career progress.**
    - Strongly agree
    - Agree

- Neutral
- Disagree
- Strongly Disagree

**15. I attribute the peer mentorship groups (E.g. Emerging scientist, social scientist, and PhD club and senior scientist forum) received at IDI to my research career progress.**

- Strongly agree
- Agree
- Neutral
- Disagree
- Strongly Disagree

**16. I attribute the Grants support (Writing, finance, grants, and research management) received at IDI to my research career progress**

- Strongly agree
- Agree
- Neutral
- Disagree
- Strongly Disagree

**17. The Host institution (IDI) provides sufficient support (Space, patients cohort data, procurement and research lab) for my research and career progress.**

- Strongly agree
- Agree
- Neutral
- Disagree
- Strongly Disagree

**18. I received support about research systems (Orientation, data sharing templates, Contracts and regulatory) at IDI for my research studies and progress**

- Strongly agree
- Agree
- Neutral
- Disagree
- Strongly Disagree

THE AIDS SUPPORT ORGANISATION
28 JAN 2022
Approved, Valid Until:..........
RESEARCH ETHICS COMMITTEE

**19. The learning opportunities such as Soft skills training, Short courses (online/offsite/onsite), John Hopkins Summer Institute and Professor in Residence have had the greatest impact on your success in my research and career progress**

- Strongly agree
- Agree
- Neutral
- Disagree
- Strongly Disagree

**20. Research dissemination platforms such as Research Forum and journal club have had the greatest impact on my research and career goals.**

- Strongly agree
- Agree
- Neutral
- Disagree
- Strongly Disagree

**21. The Scholar Evaluation sessions offered by the unit (Bi – annual evaluations and quarterly reports) contribute greatly to my research and career progress.**

- Strongly agree
- Agree
- Neutral
- Disagree
- Strongly Disagree

22. **The Capacity Building Unit model used to nurture independent investigators at IDI is relevant to my research and training needs**

- Strongly agree
- Agree
- Neutral
- Disagree
- Strongly Disagree

23. **The Capacity Building Unit model used to nurture independent investigators at IDI is effective for my research and training needs**
Strongly agree

- Agree
- Neutral
- Disagree
- Strongly Disagree

24. **The Capacity Building Unit model used to nurture independent investigators at IDI is efficient for my research and training needs**

- Strongly agree
- Agree
- Neutral
- Disagree
- Strongly Disagree

THE AIDS SUPPORT ORGANISATION
28 JAN 2022
Approved, Valid Until:..........
RESEARCH ETHICS COMMITTEE

**25. Capacity Building Unit model used to nurture independent investigators at IDI is sustainable.**

- Strongly agree.
- Agree
- Neutral

- Disagree
- Strongly Disagree

**26. The Capacity Building Unit model used to nurture independent investigators at IDI is consistent**

- Strongly agree
- Agree
- Neutral
- Disagree
- Strongly Disagree

**27. Capacity Building Unit model used to nurture independent investigator is coherent and well-coordinated.**

- Strongly agree
- Agree
- Neutral
- Disagree
- Strongly Disagree

## SCHOLAR CHALLENGES ENCOUNTERED

**28. I encountered a lot of scholar related challenges before joining the Capacity Building Unit at IDI.**

- Strongly agree
- Agree
- Neutral
- Disagree
- Strongly Disagree

**29. I am encountering a lot of scholar related challenges while under the Capacity Building unit at IDI.**

- Strongly agree
- Agree
- Neutral
- Disagree
- Strongly Disagree

THE AIDS SUPPORT ORGANISATION
28 JAN 2022
Approved, Valid Until:..........
RESEARCH ETHICS COMMITTEE

## UNIT VALUES, GAPS AND RECOMMENDATIONS.

**30. The Capacity-building unit is meeting its objectives and has no gaps to work on for improvement.**

- Strongly Agree
- Agree
- Neutral

- Disagree
- Strongly Disagree

**31. The Capacity-building unit is meeting its objectives but has gaps to work on for improvement.**

- Strongly Agree
- Agree
- Neutral
- Disagree
- Strongly Disagree

**32. The unit has major gaps and needs to rethink its programmatic activities**

- Strongly Agree
- Agree
- Neutral
- Disagree
- Strongly Disagree

## POST TRAINING ATTITUDES AND BELIEFS (SPECIFICALLY FOR ALUMNI)

**33. I identify myself as a trainee of the IDI Capacity Building Unit**

- Strongly Agree
- Agree
- Neutral
- Disagree
- Strongly Disagree

**34. My expectations are being met by the unit activities and programs by the Capacity Building Unit at IDI**

- Strongly Agree
- Agree
- Neutral
- Disagree
- Strongly Disagree

THE AIDS SUPPORT ORGANISATION
28 JAN 2022
Approved, Valid Until:..........
RESEARCH ETHICS COMMITTEE

**35. I am able to achieve my set goals with the support provided by the Capacity Building Unit at IDI**

- Strongly Agree
- Agree

- Neutral
- Disagree
- Strongly Disagree

**36. My affiliation with IDI Capacity Building Unit has/ will open more employment and career advancement opportunities**

- Strongly Agree
- Agree
- Neutral
- Disagree
- Strongly Disagree

**37. Studentship duration provided by the unit is adequate for me to achieve my set targets.**

- Strongly Agree
- Agree
- Neutral
- Disagree
- Strongly Disagree

**38. I would highly recommend this program to all scholars.**

- Strongly Agree
- Agree
- Neutral
- Disagree
- Strongly Disagree

THE AIDS SUPPORT ORGANISATION
28 JAN 2022
Approved. Valid Until..........
RESEARCH [illegible]
