# Supplementary material for: Evaluating the feasibility and effectiveness of a capacity-building model to nurture junior independent clinical research investigators in Uganda
Source: PLoS One. 2026 Jul 24;21(7):e0335299. doi: 10.1371/journal.pone.0335299 (PMC13399504; doi:10.1371/journal.pone.0335299)
Supplement: S2 File — (PDF) [file pone.0335299.s002.pdf]

# SURVEY QUESTIONNAIRE FOR IDI CAPACITY BUILDING UNIT ALUMNI

## EVALUATING THE CAPACITY BUILDING MODEL USED TO NURTURE INDEPENDENT RESEARCH INVESTIGATORS AT THE INFECTIOUS DISEASES INSTITUTE, UGANDA

## SOCIO-DEMOGRAPHICS.

**For questions 1-5, please select all that apply**

1. **Age**

   - o 18-39
   - o 40-59
   - o 60+

2. **Gender**

   - o Male
   - o Female

THE AIDS SUPPORT ORGANISATION
28 JAN 2022
Approved, Valid Until:..........
RESEARCH ETHICS COMMITTEE

3. **Marital Status**

   - o Single (Never Married before)
   - o Married
   - o Divorced
   - o Widowed
   - o Others please specify: ..........................................................

4. **What is your Level of Experience?**

   - o 1 year
   - o 2-4 years
   - o 5-7 years
   - o 8+ years

5. **Job security; Please select all that apply**

   - o Short-term contract
   - o Permanently employed
   - o Volunteer
   - o Other

## ACADEMIC, PROFESSIONAL ENGAGEMENTS AND ACHIEVEMENTS

| Attribute | Before joining Capacity Building Unit at IDI | After leaving the Capacity Building Unit at IDI |
|---|---|---|
| **6. What is the level of your training?** | o Masters<br>o PhD | o Masters<br>o PhD |

| | | |
|---|---|---|
| | o Post Doc | o Post Doc |
| **7. Which of the options best describes your profession (please select all that apply)?** | o Physician<br>o Medical Officer<br>o Pharmacist<br>o Nursing Officer<br>o Laboratory Personnel<br>o Social scientist<br>o General services<br>o Other (Specify) | o Physician<br>o Medical Officer<br>o Pharmacist<br>o Nursing Officer<br>o Laboratory Personnel<br>o Social scientist<br>o General services<br>o Other (Specify) |
| **8. What activities have you been engaged in? (Please select all that apply)** | o Teaching activities<br>o Clinical activities<br>o Research activities<br>o Leadership and administrative roles<br>o Other activities(Specify) | o Teaching activities<br>o Clinical activities<br>o Research activities<br>o Leadership and administrative roles<br>o Other activities(Specify) |
| **9. What is the best description of your work place, please select all that apply and indicate if it is a public or private sector?** | o Hospital<br>o Research based institution<br>o University<br>o Other (Specify) | o Hospital<br>o Research based institution<br>o University<br>o Other (Specify) |
| **10. How many students have you mentored?** | o None<br>o 1<br>o 2-5<br>o 6-10<br>o 11+ | o None<br>o 1<br>o 2-5<br>o 6-10<br>o 11+ |
| **11. How many publications have you made?** | o None<br>o 1<br>o 2-5 | o None<br>o 1<br>o 2-5 |

THE AIDS SUPPORT ORGANISATION
28 JAN 2022
Approved, Valid Until:..........
RESEARCH ETHICS COMMITTEE

| | | |
|---|---|---|
| | o 6-10<br>o 11+ | o 6-10<br>o 11+ |
| **12. Many grants have you written and won successfully?** | o None<br>o 1<br>o 2-5<br>o 6-10<br>o 11+ | o None<br>o 1<br>o 2-5<br>o 6-10<br>o 11+ |

**SCIENTIFIC SUPPORT**

**13. I attribute Individual mentorship and supervision received from the unit to the success of my research and career advancement.**

- o Strongly agree
- o Agree
- o Neutral
- o Disagree
- o Strongly Disagree

**14. I attribute the learning opportunities received from the unit (Short courses, soft skills) to the success of my research and career advancement.**

- o Strongly agree
- o Agree
- o Neutral
- o Disagree
- o Strongly Disagree

**15. I attribute the peer mentorship groups provided by the unit (E.g. Emerging scientist, social scientist, and PhD club and senior scientist forum) to the success of my research and career advancement.**

- o Strongly agree
- o Agree
- o Neutral
- o Disagree
- o Strongly Disagree

**16. I attribute the Grants support (Writing, finance, grants, and research management) received to the success of my research and career advancement.**

- o Strongly agree
- o Agree
- o Neutral
- o Disagree
- o Strongly Disagree

THE AIDS SUPPORT ORGANISATION
28 JAN 2022
Approved, Valid Until:..........
RESEARCH ETHICS COMMITTEE

17. **IDI as a host institution provided sufficient support (Space, patients cohorts data, procurement, and research lab) for my research studies**
    - o Strongly agree
    - o Agree
    - o Neutral
    - o Disagree
    - o Strongly Disagree
18. **I received support about Research systems at IDI (Orientation, data sharing templates, Contracts and regulatory) for my studies**
    - o Strongly agree
    - o Agree
    - o Neutral
    - o Disagree
    - o Strongly Disagree
19. **Learning opportunities such as Soft skills training, Short courses (online/offsite/onsite), John Hopkins Summer Institute and Professor in Residence programs had the greatest impact on my research and career success.**
    - o Strongly agree
    - o Agree
    - o Neutral
    - o Disagree
    - o Strongly Disagree
20. **Research Dissemination platforms such as Research Forum and journal club had the greatest impact on my research and career success.**
    - o Strongly agree
    - o Agree
    - o Neutral
    - o Disagree
    - o Strongly Disagree
21. **The evaluation sessions such as Bi – annual evaluations and quarterly reports submissions were useful to my research and career advancement.**
    - o Strongly agree
    - o Agree
    - o Neutral
    - o Disagree
    - o Strongly Disagree

22. **The Capacity Building Unit model used to nurture an independent investigator at IDI was relevant for my research training needs**
    - o Strongly agree
    - o Agree
    - o Neutral
    - o Disagree
    - o Strongly Disagree

THE AIDS SUPPORT ORGANISATION
28 JAN 2022
Approved, Valid Until:..........
RESEARCH ETHICS COMMITTEE

23. **The Capacity Building Unit model used to nurture independent investigators was effective for my research and training needs**

Strongly agree

- Agree
- Neutral
- Disagree
- Strongly Disagree

24. **The Capacity Building Unit model used to nurture independent investigators at IDI was efficient for my research training and needs**

- Strongly agree
- Agree
- Neutral
- Disagree
- Strongly Disagree

25. **The Capacity Building Unit model used to nurture independent investigators at IDI is sustainable.**

- Strongly agree.
- Agree
- Neutral
- Disagree
- Strongly Disagree

26. **The Capacity Building Unit model used to nurture independent investigators at IDI is consistent**

- Strongly agree
- Agree
- Neutral
- Disagree
- Strongly Disagree

27. **The Capacity Building Unit model used to nurture independent investigators at IDI is coherent and well-coordinated.**

- Strongly agree
- Agree
- Neutral
- Disagree
- Strongly Disagree

## SCHOLAR CHALLENGES ENCOUNTERED

28. **I encountered scholar related challenges before joining the IDI capacity Building unit (pre-training).**

- Strongly agree
- Agree
- Neutral

THE AIDS SUPPORT ORGANISATION
28 JAN 2022
Approved, Valid Until:..........
RESEARCH ETHICS COMMITTEE

- o Disagree
- o Strongly Disagree

**29. I encountered scholar related challenges after joining the IDI capacity Building unit (In-training).**

- o Strongly agree
- o Agree
- o Neutral
- o Disagree
- o Strongly Disagree

**30. I encountered scholar related challenges (post-training) after graduating for IDI capacity Building unit.**

- o Strongly Agree
- o Agree
- o Neutral
- o Disagree
- o Strongly Disagree

## UNIT VALUES, GAPS AND RECOMMENDATIONS.

31. **The Capacity-building unit is meeting its objectives and has no gaps to work on for improvement.**

- o Strongly Agree
- o Agree
- o Neutral
- o Disagree
- o Strongly Disagree

32. **The Capacity-building unit is meeting its objectives but has gaps to work on for improvement.**

- o Strongly Agree
- o Agree
- o Neutral
- o Disagree
- o Strongly Disagree

**33. The unit has major gaps and needs to rethink its programmatic activities**

THE AIDS SUPPORT ORGANISATION
28 JAN 2022
Approved, Valid until:..........
RESEARCH ETHICS COMMITTEE

- o Strongly Agree
- o Agree
- o Neutral
- o Disagree
- o Strongly Disagree

**34. I identify myself as an alumni of the IDI Capacity Building Unit**

- Strongly Agree
- Agree
- Neutral
- Disagree
- Strongly Disagree

35. **My expectations were met by the Capacity building unit activities and programs**
    - Strongly Agree
    - Agree
    - Neutral
    - Disagree
    - Strongly Disagree
36. **I was able to achieve my career set goals while receiving support from the Capacity Building Unit**
    - Strongly Agree
    - Agree
    - Neutral
    - Disagree
    - Strongly Disagree
37. **My affiliation with IDI Capacity Building Unit opened more employment and career advancement opportunities for me**
    - Strongly Agree
    - Agree
    - Neutral
    - Disagree
    - Strongly Disagree
38. **The studentship duration offered by Capacity Building Unit was adequate for me to achieve my set targets.**
    - Strongly Agree
    - Agree
    - Neutral
    - Disagree
    - Strongly Disagree

THE AIDS SUPPORT ORGANISATION
TASO
28 JAN 2022
Approved. Valid Until:..........
RESEARCH ETHICS COMMITTEE

39. **I would highly recommend this program to all scholars.**
    - Strongly Agree
